# Inclusion of adolescent girls in HIV prevention research – an imperative for an AIDS-free generation

**DOI:** 10.7448/IAS.17.1.19075

**Published:** 2014-03-08

**Authors:** Quarraisha Abdool Karim, Rachael Dellar

**Affiliations:** CAPRISA, Nelson R Mandela School of Medicine, University of KwaZulu-Natal, Durban, South Africa

Recent scientific advances centred on the use of anti-retrovirals (ARVs) – both prophylactically to prevent HIV acquisition (pre-exposure prophylaxis, or PrEP) and for treatment to minimize onward transmission (treatment as prevention, or TasP) – have led to a new-found optimism for control of the HIV/AIDS epidemic and the possibility of creating an “AIDS-free generation” [1]. In order to translate this optimism into reality, large and sustained reductions in incident HIV infections are required. Several models have projected that with substantial programmatic scale-up of the new prevention agenda, such requirements can be satisfied [2]. However, these models typically assume a uniform efficiency for interventions in reducing incident infections across populations, and neglect to consider their current unavailability to a key population driving the epidemic at its epicentre: adolescent girls.

In sub-Saharan Africa, which continues to bear a disproportionate burden of new HIV infections, almost a third of new infections occur in young women aged between 15 and 24 years [3]. In this region, a defining characteristic of the epidemic is the age-sex distribution in HIV acquisition, wherein women acquire HIV infection about five to seven years earlier than their male peers, often synonymously with sexual debut [4]. Particularly in southern Africa, this age-sex difference in HIV acquisition rates has contributed to unprecedentedly high incidence rates in adolescent girls, and continues to sustain the epidemic. In South Africa, more than 20% of young pregnant women aged 15–24 years attending antenatal clinics are HIV positive, and more than three-quarters of HIV-positive young people aged 15–24 years are women [5]. The reasons for such high rates of new infections are complex and are compounded by a number of structural, social and biological factors; however, what is clear is that an AIDS-free generation cannot be realized unless new infections in adolescent girls are eliminated. This is a public health imperative.

The HIV prevention interventions available to this population are abstinence, promotion of condom use, behaviour change (including delay of sexual debut) and conditional cash transfers to encourage high school completion. However, these interventions are highly variable in their robustness and, given the underlying gender-power dynamics in the sub-Saharan African setting, they are likely to be of limited immediate benefit for the most vulnerable young women. Moreover, while community-based interventions and school-based education programmes to address the underlying economic and social vulnerability of adolescent girls should be encouraged, this is a substantial task that will potentially require decades of concentrated action, during which time adolescent girls will continue to become infected.

Furthermore, immediate measures to protect adolescent girls are urgently needed. However, new prevention advances that may have the potential to afford such immediate protection are currently unavailable to adolescent girls despite potentially substantial population- and individual-level benefit because of exclusion of young women under 18 years of age from trial participation. There is thus an urgent need to validate the safety and efficacy of existing technologies for use in adolescents, which may take several years to accomplish. In the interim, the need for safe and efficacious prevention options is immense and growing. As this gap is filled, it is critical not to increase the deficit, and future prevention trials, including vaccine trials, should include adolescent girls from the outset.

As important as the inclusion of adolescent girls in prevention trials is the inclusion of adolescent girls in treatment trials to address the needs of the significant proportion of adolescents who are already living with HIV. Indeed, in Lesotho, an estimated 20% of all young people aged 15–24 years are infected with HIV, and adolescents (10–19 years) are the only age group in which AIDS deaths have risen between 2001 and 2012 [6].

The epidemiological evidence for the high risk and burden of HIV in adolescent girls is overwhelming and, together with the lack of evidence of protection from behavioural interventions, has prompted this and other calls for their inclusion in both biomedical prevention and treatment trials. The recent establishment of the Lancet Adolescent Commission is in recognition of the global importance of this key population [7]; yet in the absence of evidence-based HIV prevention approaches, it may have limited impact. To facilitate adolescent participation in clinical and preventive trials, by extension we are also calling for increased research on key parameters in adolescents and for a more enabling ethico-legal framework conducive to adolescent recruitment. Currently, moral and judgemental values override the epidemiological evidence in decisions relating to the autonomous inclusion of adolescents in research, notwithstanding consensus documents generated by UNAIDS on the importance of the inclusion of adolescents in HIV preventive vaccine trials [8]; the dearth of data on adolescents is lagging behind the policy and practice discourse. Consolidation of available data from treatment provision and sexual reproductive health service provision services targeted at African adolescents will be an important first step in enhancing our understanding of pathogenesis and disease progression in this population to inform prevention and treatment efforts.

A major barrier to the inclusion of adolescents in research is restrictive ethico-legal frameworks [9]. Ethics guidelines and legislation are based on historical protection of minors who are perceived to be incapable of providing autonomous consent. In communities where, prior to ARV treatment access, large numbers of adults were decimated by AIDS, there are now large numbers of child-headed households. Here, adolescents take responsibility for themselves and their younger siblings, and show considerable maturity; yet they are unable to independently take steps to participate in HIV research due to lack of parental consent. Further barriers to obtaining parental consent in the realities of sub-Saharan African settings result from high levels of migration for employment, which perturb traditional family structures, and the typical tendency of not formally establishing legal guardianship in the absence of parents.

Beyond barriers to participation, stipulating the need for parental consent in current guidelines may inadvertently be placing an already vulnerable population at risk of harm. Indeed, non-autonomous participation of adolescents may necessitate breaching the confidentiality of research participants, particularly with regard to parental disclosure of sexual activity and/or HIV status. Such breaches of confidentiality could place participants at potential risk of harm given that discrimination, violence and social ostracism are commonly experienced by those whose HIV status is disclosed to family or community members in this setting. Even for HIV-negative participants, there may be a risk of discrimination as involvement in preventive trials carries implications for their levels of sexual activity.

The need to include adolescents in HIV research must be carefully balanced against the need to protect them and, clearly, if autonomous participation for adolescents is widely adopted, it should be carefully regulated to protect adolescents from any harm associated with research, particularly relating to potential reactions to discovery of HIV-positive status. Indeed, adolescents seeking autonomous participation should be able to demonstrate appropriate cognitive and emotional maturity during informed consent procedures and throughout the trial, for example, by successful completion of a cognitive and emotional maturity assessment. Other methods of minimizing harm could include the establishment of committees of community members capable of overseeing consent procedures. These are complex challenges with no easy answers, but they must be proactively and pragmatically addressed as we consider the cost and harm of inaction.

To conclude, the unprecedentedly high HIV incidence rates in adolescent girls make their inclusion in HIV research a public health imperative, both in terms of their own protection or treatment access, and for realizing the goal of an “AIDS-free generation.” To this end, a concerted effort is required by all stakeholders as a matter of urgency to ensure a safe and enabling framework for participation of adolescents in HIV research to ultimately facilitate access to much-needed evidence-based HIV prevention and treatment services. Young women have a right to remain HIV uninfected, and allowing this right to be exercised has both individual and population benefits. In the context of the HIV epidemic, autonomous decision making by adolescents could enable this process and, in future, offer some protection to vulnerable women that science cannot yet afford them.
